# The European Union Emissions Trading System reduced CO_2_ emissions despite low prices

**DOI:** 10.1073/pnas.1918128117

**Published:** 2020-04-06

**Authors:** Patrick Bayer, Michaël Aklin

**Affiliations:** ^a^School of Government and Public Policy, University of Strathclyde, Glasgow G1 1QX, United Kingdom;; ^b^Department of Political Science,University of Pittsburgh, Pittsburgh, PA 15260

**Keywords:** carbon markets, EU ETS, policy evaluation, synthetic control

## Abstract

International carbon markets are an appealing and increasingly popular tool to regulate carbon emissions. By putting a price on carbon, carbon markets reshape incentives faced by firms and reduce the value of emissions. How effective are carbon markets? Observers have tended to infer their effectiveness from market prices. The general belief is that a carbon market needs a high price in order to reduce emissions. As a result, many observers remain skeptical of initiatives such as the European Union Emissions Trading System (EU ETS), whose price remained low (compared to the social cost of carbon). In this paper, we assess whether the EU ETS reduced ${CO}_{2}$ emissions despite low prices. We motivate our study by documenting that a carbon market can be effective if it is a credible institution that can plausibly become more stringent in the future. In such a case, firms might cut emissions even though market prices are low. In fact, low prices can be a signal that the demand for carbon permits weakens. Thus, low prices are compatible with successful carbon markets. To assess whether the EU ETS reduced carbon emissions even as permits were cheap, we estimate counterfactual carbon emissions using an original sectoral emissions dataset. We find that the EU ETS saved about 1.2 billion tons of ${CO}_{2}$ between 2008 and 2016 (3.8%) relative to a world without carbon markets, or almost half of what EU governments promised to reduce under their Kyoto Protocol commitments. Emission reductions in sectors covered under the EU ETS were higher.

## Significance

International carbon markets are an appealing and increasingly popular tool to regulate carbon emissions. They put a price on carbon emissions and make pollution less attractive for regulated firms. However, carbon markets often produce prices which are deemed too low relative to the social cost of carbon. We argue that despite low prices, carbon markets can help reduce emissions. Using a statistical model and sectoral emissions data, we find that the EU ETS, which initially regulated roughly 50% of EU carbon emissions from mainly energy production and large industrial polluters, saved more than 1 billion tons of CO_2_ between 2008 and 2016. This translates to reductions of 3.8% of total EU-wide emissions compared to a world without the EU ETS.

Strong policy action is necessary to curb greenhouse gas (GHG) emissions (1–3). Despite its shortcomings, a feature of the climate regime has been its willingness to experiment with a wide range of tools, including decentralized, market-based institutions (4). Market-based institutions rely on markets to price externalities and change the behavior of firms and individuals.

Among such institutions, the European Union’s Emissions Trading System (EU ETS) stands out as the most ambitious attempt to date to reduce carbon emissions. The EU ETS has been the EU’s flagship initiative to reach its climate targets under the Kyoto Protocol. It is a cap-and-trade system in which governments set an allowable total amount of emissions (“cap”) over a certain period and issue tradable emission permits (“trade”). These permits, which are typically good for 1 ton of ${CO}_{2}$, are the currency in carbon markets.

Carbon markets are appealing as they reduce emissions at lowest cost, at least theoretically (5). This is the main reason why they are attracting much attention from policymakers. China currently is in the midst of setting up its own national carbon market, and more than 80 countries mention carbon markets in their commitments under the 2015 Paris Agreement on Climate Change as their preferred policy instrument for reducing carbon emissions (6).

To assess the desirability of carbon markets, we estimate the effect of the EU ETS on carbon dioxide emissions. Evidence of the effectiveness of carbon markets and the EU ETS remains scarce (7, 8). For the most part, observers have been critical and point to the low market prices as a major problem. The Organisation for Economic Cooperation and Development (OECD) bemoaned low prices in a recent report over worries that cheap permits fail to incentivize polluters to reduce carbon emissions and invest in abatement technology (9). Traditionally, prices in the EU ETS were believed to be low because of the oversupply of permits and decreased demand during the financial crisis (10).

Notwithstanding that higher prices will speed up the low carbon energy transition (11), low prices can be compatible with decarbonization. This is because low carbon prices are consistent with both high supply from overallocation and low demand because of behavioral changes among regulated firms. We argue that as long as at least some firms interpret carbon regulation through the EU ETS as a credible signal that governments will impose serious costs on carbon emissions in the long run, even low prices today should result in observable carbon reductions. Several studies find that low EU ETS prices have not prevented at least some regulated firms to invest in abatement technologies and reduce the carbon intensity of their production (8, 12–14).

Building on this literature, we draw on sectoral emissions data to answer whether carbon markets are effective in international settings, which are more difficult to regulate than domestic issues. We assess the effectiveness of the EU ETS with the help of a statistical technique. The generalized synthetic control approach (15) allows us to estimate counterfactual emissions paths, which we compare to actual emissions to recover reliable estimates of the effect of the EU ETS. In contrast to widespread skepticism, our estimates suggest that the EU ETS saved about 1.2 billion tons of ${CO}_{2}$ from 2008 to 2016, roughly 3.8% relative to total emissions over this period. These reductions amount to almost half of the EU-wide Kyoto target and are driven by sectors covered under the EU ETS, such as energy production and large industrial polluters. They emitted 11.5% (95% confidence interval [−16.9%, −5.4%]) less than they would have in a world without the EU ETS.

## Background on EU Carbon Markets

After the ratification of the Kyoto Protocol in 2002, EU member states were tasked to implement treaty commitments (16). The Protocol gave considerable freedom about how to achieve the EU-wide common target—an 8% reduction of GHG emissions by 2012 relative to 1990 levels. Already in 1999, EU member states agreed on the internal, by-country distribution of carbon reductions (17), but how best to reduce emissions remained contested. In 2003, member states agreed to a supranational emissions trading scheme, the EU ETS (Directive 2003/87/EC). According to initial rules, each member state had to submit National Allocation Plans, which detailed a country-wide reduction target together with a list of regulated installations. After the approval of these plans by the European Commission, installations received permits that could be traded. By the end of April each year, installations that hold too few permits to cover their emissions need to buy additional permits from the market or pay a penalty of €40 (2005 to 2007) or €100 (since 2008) for each ton of carbon they fall short.

The EU ETS started in 2005 and operates in phases. The first phase, from 2005 to 2007, was a pilot to get the system up and running (18). The second phase covered the Kyoto Protocol commitment period, 2008 to 2012. Finally, the third, currently ongoing phase started in 2013 and will last until 2020. During the first phase, about 12,000 installations received permits to emit roughly 2.2 billion tons of ${CO}_{2}$ across the then 25 EU members, covering almost 50% of the EU’s total ${CO}_{2}$ emissions (19).

**Fig. 1.**
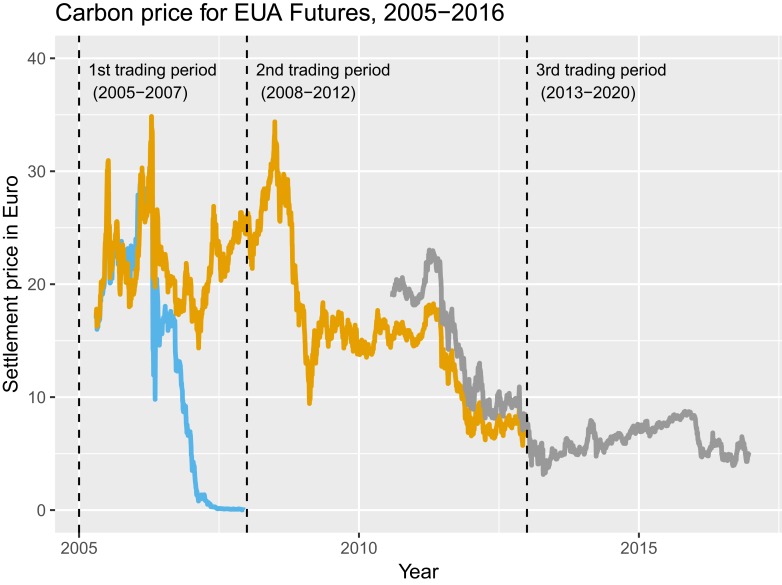
Cost of 1 ton of ${CO}_{2}$ in the EU ETS, 2005 to 2016. The plot shows EU Allowance (EUA) settlement prices in future markets for permits of December 2007 (blue), December 2012 (yellow), and December 2016 (gray) maturities. The dashed vertical lines mark trading periods. As permits could not be carried forward from the first into the second trading period, prices dropped to 0 by December 2007. Source: Intercontinental Exchange (ICE Futures Europe), accessed through Quandl (CZ2007, CZ2012, and CZ2016).

The overall assessment of the EU ETS is mixed (8). Initially, the lack of reliable baseline data troubled the EU ETS and encouraged regulated emitters to inflate their emissions (20). Carbon prices, shown in Fig. 1, remained below levels generally believed to be needed to curb emissions (21–23), which fueled concern about the usefulness of the policy. Low prices result from one or several of the following reasons (21): first, demand for permits was low because of the economic crisis (5, 24); second, competing policies, such as renewable and efficiency targets, which also aim at reducing carbon emissions deflate demand (25–27); and third, permits from international offset schemes like the Clean Development Mechanism caused EU ETS prices to plummet (28, 29).

All these studies share skepticism for the EU ETS and argue that low prices undermine the proper functioning of carbon markets. Despite these concerns, others tried to identify the causal effect of the EU ETS on emission reductions. The challenge here consists in knowing what emission levels would have been in the absence of the EU ETS (30): the “counterfactual is not observed and never will be. It can only be estimated, but there are better and worse estimates” (ref. 31, p. 277). Early studies find annual reductions of 50 to 100 Mt (2 to 5%) in 2005/2006 (31). This estimate happens to be numerically close to ours, yet is based on tentative EU-level data and only covers the first 2 y of the EU ETS, while our statistical approach and granular data allow us to disaggregate estimates by country and sector and over a much longer time period.

Concerns over low permit prices triggered the European Commission to reform the EU ETS recently. In a first step, the auctioning of 900 million permits was postponed to 2019/2020 to address the imbalance of demand and supply (32). As a result, prices increased fourfold from €5 for most of the third trading period to about €20 in the latter half of 2018. The fourth trading period from 2021 to 2030 will also further strengthen reduction targets and limit the use of international carbon credits. The most notable reform, which the European Commission thinks will restore “normal functioning of the EU ETS” (ref. 33, p. 92) despite academic skepticism (34, 35), is the introduction of the Market Stability Reserve. It acts like a central bank and either injects or withdraws liquidity, in the form of permits, into the market to stabilize prices.

## The Limits of Price as a Heuristic for Effectiveness

Several observers use carbon prices as the first go-to gauge to assess how well carbon markets work. Explicitly or implicitly, low prices are assumed to mean that carbon markets are underperforming. In general, prices have been used as a heuristic to assess policy effectiveness on two grounds. First, the importance of carbon pricing has often been studied through the lens of integrated assessment models (36, 37). These models seek to monetarize future damages from emitting carbon as the social cost of carbon, which is then taken as the cost at which carbon should be priced. Seeing a mismatch between the social cost of carbon and market prices, many are tempted to conclude that market prices are not high enough to discourage emissions.

This is, however, not an entirely accurate reading of what these models say. The social cost of carbon is useful as a measure of welfare loss from carbon emissions, but we cannot infer from it that the carbon price needs to be equal to the social cost of carbon to deter any emissions at all. Notwithstanding that higher prices incentivize more investment in technologies to combat climate change (11), prices in the short run are unimportant if low carbon investments promise a future comparative advantage over slowly decarbonizing competitors (38).

Second, low prices are linked to oversupply of carbon permits (39, 40). Governments tended to issue too many permits to protect their industries from costly carbon regulation. Initially, this resulted in downward pressure on prices (41, 42), while more recently, low prices were mainly associated with weak demand due to the financial crisis and market imperfections (35).

Oversupply undoubtedly leads to low prices, but the reverse does not have to be true. Prices can also be low because the demand for carbon permits decreases. Low prices alone are therefore inconclusive about the reason for why they are low. For us, the more important question is whether declining demand for permits in the EU ETS is plausible. We argue that there is a very realistic chance for this: the EU ETS is anchored in European law, and the European Commission seeks to achieve net carbon neutrality in 2050, so it seems unreasonable for regulated firms to expect that the EU ETS will go away any time soon. Greater scarcity in European carbon markets as a result of recent reforms and the introduction of the Market Stability Reserve made prices spike to €25 in early 2020, which is consistent with our argument that the EU ETS will persist and prices will rise.

Under these conditions, carbon abatement makes sense even if current market prices are low. Manufacturing firms in Germany and France, for instance, reduced their ${CO}_{2}$ emissions by 15 to 20% already in the early 2007 to 2010 years of the scheme (12, 13). The EU ETS triggered low carbon innovation of roughly 10% (43), and firm representatives report that it affects their long-term investment strategies (14). Despite low prices today, the EU ETS can effectively reduce emissions by credibly signaling much increased cost in the future.

## Measuring the Effectiveness of the EU ETS

Evaluating the effectiveness of the EU ETS should hence not rely on market prices but rather assess whether the policy caused emissions to go down. Methodologically, this is a difficult task as it requires us to compare actual emission levels under the EU ETS with emission levels which we would have seen had the EU ETS never been introduced (44, 45). Since these so-called counterfactual emissions cannot be observed, we use a statistical model to estimate them (18).

For this, we rely on a special type of synthetic control method (*Materials and Methods*). In its standard form, synthetic control is a weighting approach (46, 47): it weights control group units, so that their weighted average mimics the treated group as much as possible. Since only data from before policy implementation are used to construct weights, any differences in outcomes between the (weighted) control and treatment group after policy implementation must be due to the policy itself. This approach is powerful for assessing policy effectiveness in a single country. However, to evaluate policies, such as the EU ETS, which are introduced in multiple countries simultaneously, we instead resort to the generalized synthetic control method (15).

Similar in spirit, the generalized synthetic control method still uses a reweighting scheme to construct the counterfactual but estimates a statistical (linear interactive fixed effects) model before assigning weights. This estimation strategy makes a direct interpretation of weights difficult but allows us to explicitly model how structural factors, such as economic output or growth in renewable energy production, affect carbon emissions. With the inclusion of appropriate control variables, our linear model can additionally pick up on other carbon legislation and policies, such as carbon taxes. The interactive fixed effects finally capture unobserved time-varying confounding from, for instance, different effects of the 2007/2008 financial crisis on European economies and their emissions (48).

In our case, we use the synthetic control method to predict counterfactual emissions for sectors covered under the EU ETS (ETS sectors)—i.e., we estimate what ${CO}_{2}$ emissions would have been without the EU ETS policy—from observable emissions in those sectors that are not covered under it (non-ETS sectors). Importantly, this means that our estimate of EU ETS effectiveness is not just the difference between emissions in ETS and non-ETS sectors; instead, counterfactual emissions (control group) against which we compare actual emissions from ETS sectors (treatment group) are estimated from, and are hence not simply identical to, non-ETS sector emissions. This estimation part in our empirical strategy is critical because it ensures that counterfactual and ETS sector emissions are comparable as much as possible, even if emission levels of ETS and non-ETS sectors differ.

Applying this empirical strategy requires us to know, for each country and both before and after the adoption of the EU ETS, how many emissions were emitted by ETS and non-ETS sectors, respectively. Once carbon markets became operative in 2005, this is easy as ETS sector emissions data are available from the official EU Transaction Log (EUTL) of the EU ETS (49). For the years before the EU ETS was launched, this poses, however, a nontrivial data challenge. Total emissions data since 1990 exist from national communications to the United Nations (UN) (50), but it is unclear what share of these total emissions will be covered under the future EU ETS.

We resort to sectoral emission breakdowns to address this problem. Specifically, for the years 2005 to 2016, when we have both EU ETS and UN data, we identify sectors and groups of sectors whose emissions are as close to each other as possible. In a second step, we then use UN emissions data from this matched sector as the values for this sector’s emissions in 1990 to 2004, which will come under EU ETS carbon market regulation in the future. For instance, for energy sector emissions in our dataset (51), we first match emissions from fuel combustion and oil refining from the EU ETS data (EU ETS activity codes 20 and 21) with emissions from energy industries from the UN data (UN category 1.A.1). In constructing matching sectors, we follow the UN guidelines on sectoral correspondence across both datasets and, where discretion applies, maximize the numerical fit in our data. Then, we use UN emissions from UN category 1.A.1 as the (pretreatment) EU ETS emissions for the (treated) energy sector, which will be covered under the EU ETS in the future. Our so-created dataset provides sector-level emissions covered by the EU ETS as well as total emissions covered by the EU ETS by summing up emissions across all ETS sectors. Total EU ETS emissions, which we use for our main analysis below, match UN emissions with high accuracy of 97.2% (*SI Appendix*, Table S1), while sector-level matches, especially for the chemical industry, are less accurate (*SI Appendix*, Table S2). Interactive fixed effects do, however, absorb some of these level differences.

To build our counterfactual using the generalized synthetic control method, we model countries’ carbon emissions as a function of logged gross domestic product (GDP) and logged GDP squared as main variables. Albeit simple, the flexible, quadratic functional form together with the interactive fixed effects allows the model to capture variability in the data well. We also show that more fully specified models, including controls for renewable electricity production and climate-related policies, produce similar results (*SI Appendix*, Table S3). We then recover estimates of the effect of the EU ETS by comparing total emissions covered under the EU ETS to estimated counterfactual emissions. Averaging across countries and years results in the average treatment effect on the treated (ATT) of the EU ETS. Since our comparisons are within country, our estimates are less sensitive to concerns of outsourcing emissions to unregulated countries and are also robust to changes in input prices, such as for oil. Our analysis does not allow for conclusions about the effect of the EU ETS on worldwide carbon emissions, but it estimates the effect of the EU ETS on those European countries which adopted carbon market regulation with reasonable reliability.

## Results

Table 1 shows our main results. We report results separately for 2005 and 2008 because both are plausible starting dates for European carbon regulation. The pilot period of the EU ETS started in 2005, whereas 2008 marks the beginning of the second trading period which was aligned with the Kyoto Protocol’s commitment period (2008 to 2012). Because of increased stringency in 2008, we expect stronger reduction effects (12).

**Table 1.** Effect of the EU ETS on ${CO}_{2}$ emissions

	Generalized synthetic control	
	2005	2008
Estimate summary		
Mean	−8.1%	−11.5%
95% CI	[−13.2%, −1.7%]	[−16.9%, −5.4%]
Observations		
Full sample	1,304	1,304
Pretreatment	704	854
Posttreatment	600	450

Results are shown for two treatment years: 2005 when the pilot period started (second column) and 2008 when the second trading period started (third column). The table shows ATT estimates and bootstrapped 95% confidence intervals as well as the number of observations. Point estimates are converted into percentage point changes for substantive interpretation.

The estimates show a decrease of ${CO}_{2}$ emissions in ETS sectors of between 8.1 and 11.5% against the counterfactual. Since the counterfactual is constructed from non-ETS sector emissions, which were still regulated to some extent under the EU’s Effort Sharing Decision, our estimates are conservative as they do not compare emission reductions against a no-regulation scenario. As expected, we find that the estimates increase for 2008 as treatment year. This finding is consistent with the low effectiveness of the EU ETS during the pilot phase from 2005 to 2007 when too many permits were issued (7).

To be clear, these results do not say that carbon emissions across Europe reduced by 8.1 to 11.5% over the last decade. Reductions were much higher, of course. Instead, these reductions are estimates of additional ${CO}_{2}$ emission reductions because of carbon market regulation 1) in ETS sectors and 2) on top of the decline in emissions in non-ETS sectors. The EU ETS carbon market policy is thus associated with substantial decarbonization of 1.2 billion tons during 2008 to 2016. We cannot rule out that some of these reductions, especially in initial years, were due to cheap, “low-hanging fruit” decarbonization. One concern, then, is that the effectiveness of the EU ETS could decline as it deals with tougher cases. Against this worry, we note that there is evidence for a more systematic transformation away from carbon (52). There are several potential reasons for this, one being that a first-mover advantage encourages even firms with high abatement costs to decarbonize (38).

**Fig. 2.**
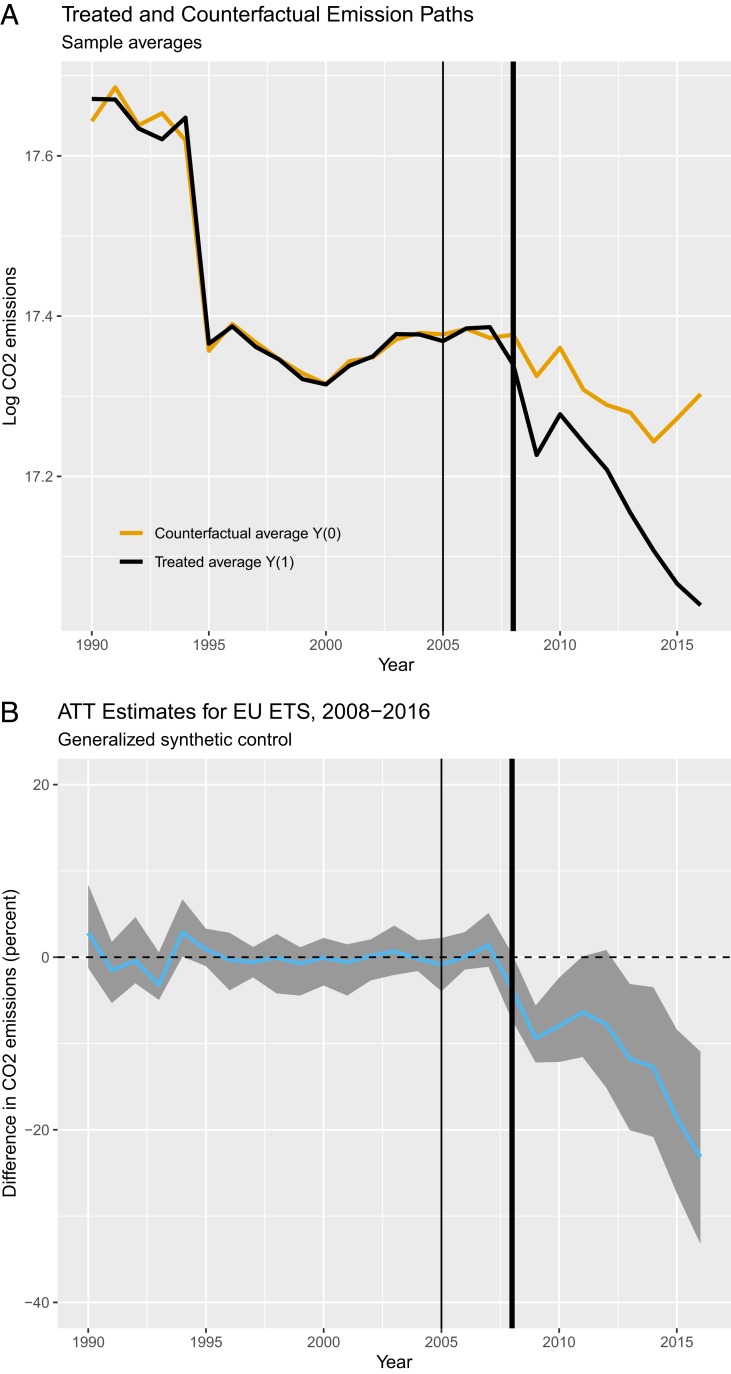
Effect of the EU ETS over time. (*Top*) The mean ${CO}_{2}$ emissions paths for actual (black line) and counterfactual (yellow line) emissions. (*Bottom*) The estimated ATT of the EU ETS (blue line) and bootstrapped 95% confidence intervals (gray area). The thin and thick black lines mark years 2005 (start of pilot period) and 2008 (start of second trading period).

Fig. 2 shows how the effect of the EU ETS evolved over time and strengthens the point that decarbonization has considerably deepened at least since 2008. Fig. 2, *Top*, shows mean emissions paths for actual emissions from ETS sectors (black line) relative to counterfactual emissions (yellow line). Emissions in both groups decline over time, but ETS sector emissions do so much faster. This captures the additional decarbonization effect of the EU ETS. It also suggests that market-based regulation phased out emissions more quickly than classical command-and-control regulation in the counterfactual case under effort sharing legislation.

Fig. 2, *Bottom*, shows the estimated ATT of the EU ETS (blue line). The gray areas are bootstrapped 95% confidence intervals. There are three takeaway messages. First, the statistical model produces a good counterfactual as it fits the pre-EU ETS trend before 2005 well. Second, the difference between actual and counterfactual emissions increases over time, so the EU ETS effect accumulates the longer the policy has been in place. Third, the mean estimate significantly breaks off from the zero line at around 2008, with the onset of the second trading period.

We report our results separately for each of the then 25 EU member states (*SI Appendix*, Fig. S1). With few exceptions, the general pattern holds across all countries: ETS sector emissions experienced an abnormal decline after 2008 when the stringency of the EU ETS policy increased considerably. We furthermore show by leaving out a country at a time that the effectiveness of the EU ETS is not driven by a single country (*SI Appendix*, Fig. S2). Simulating the start date of the EU ETS in a ±5-y window around 2008 does not reproduce our results, which suggests that the driver behind the strong decline in emissions has indeed been carbon market regulation and not some other policy that had been adopted before or after the launch of the EU ETS (*SI Appendix*, Fig. S3).

In summary, we find strong evidence that EU carbon markets have been effective despite low market prices. Importantly, our estimated emission reductions of between 8.1 and 11.5% are on top of any emission reductions from reduced economic output during the 2007/2008 financial crisis. Notwithstanding the novelty of these results, they do not imply that carbon markets are necessarily the most effective policy for a low carbon energy transition. Even though EU-wide policy options were limited due to the resistance to a carbon tax, other policies might have been more effective. However, once the EU had launched their scheme, the prospect of linking other carbon markets to the European one created immediate pull. This fast-tracked the diffusion of carbon markets globally (6). Our results shine light on the effectiveness of the EU ETS relative to the case of no carbon markets, without speculating about which other, possibly more effective policies could have been adopted in the absence of the EU ETS.

## Local and Global Emission Reductions

We use our estimates to calculate the substantive effect of the EU ETS as an overall reduction of ${CO}_{2}$ emissions by 1.2 billion tons during 2008 to 2016. Relative to the emissions covered under the EU ETS, this translates to a reduction of about 7.5% or roughly 3.8% compared to the EU’s total emissions (*SI Appendix*, Table S4). Notwithstanding variation in effectiveness across EU member states, this amount is quite sizable as it accounts for almost half of the EU governments’ commitments under the Kyoto Protocol.

While we have shown that the EU ETS contributed substantially to the EU’s efforts to decarbonize its economies despite low carbon prices, we cannot rule out that some of these reductions were achieved by moving production outside of the EU and into countries without carbon markets, which is typically referred to as carbon leakage (53, 54). If such leakage happens, the net effect of the EU ETS on global emissions must be weaker than its local effect on EU-wide emissions. Our estimate is therefore an upper bound for the global effect. Even though our analysis cannot comment on the lower bound, which would require quantifying the size of the leakage problem, our results are promising from a climatic standpoint for two reasons. First, our estimates of local effects demonstrate that the EU ETS did change emission patterns in Europe. For other countries and regions around the world, this means that introducing carbon markets, even with low prices, can be an effective decarbonization strategy. As argued above, the political will to commit to carbon markets in the long run is, however, critical for this effect to materialize.

**Fig. 3.**
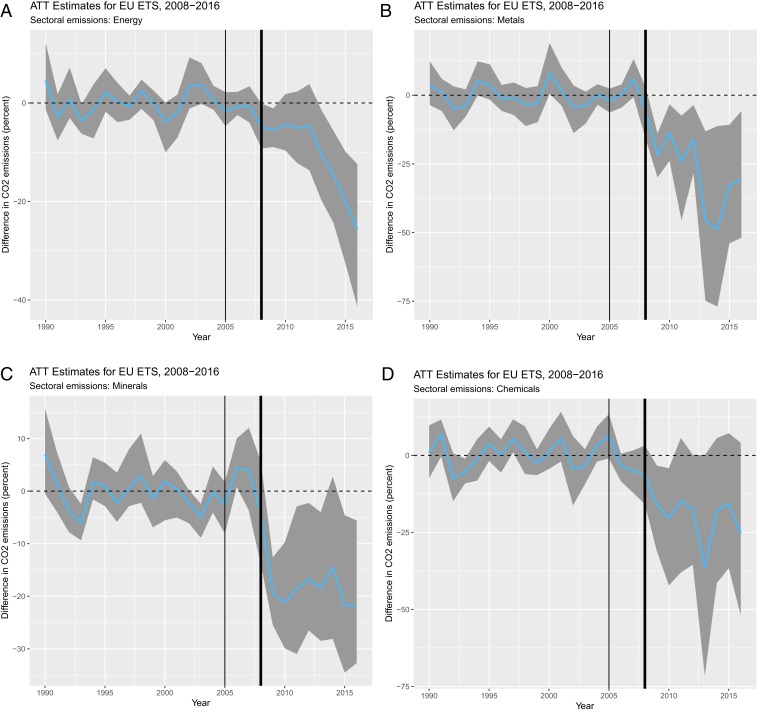
Effect of the EU ETS on different sectors covered under the EU ETS. The plots show the mean ATT estimate of the EU ETS (blue line) and bootstrapped 95% confidence intervals (gray area) for four sectors: (*Top Left*) energy, (*Top Right*) metals, (*Bottom Left*) minerals, and (*Bottom Right*) chemicals. The thin and thick black lines mark years 2005 and 2008 for the start of the EU ETS pilot period and the second trading period, respectively.

Second, we use a sector-level analysis of EU ETS effectiveness of four major industries to check that emission reductions happened across all industries. While ${CO}_{2}$ reductions in mobile sectors may be due to relocation of production facilities, reductions in immobile sectors are more likely to result in actual reductions at home rather than only the displacement of emissions. Fig. 3 shows that emissions decreased between 20 and 25% against the counterfactual in all sectors, similar to what others have found (12, 13). A robust decline in emissions in energy production, which is fairly immobile due to a large share of fixed assets, makes us the more confident that the EU ETS did not only achieve emission reductions through carbon leakage. Qualitative, firm-level evidence indicates that electricity utilities responded to the EU ETS by “embark[ing] on a strategy to decarbonize power supply in Europe by 2050” (ref. 52, p. 283). Strengthening our claim further, a placebo test reveals that transport emissions, which are not covered by the EU ETS, did not decrease at all since the introduction of EU carbon markets (*SI Appendix*, Fig. S4).

## Conclusion

More and more governments around the world have been adopting carbon markets to regulate GHG emissions (6). China is in the midst of rolling out a national emissions trading scheme, which is expected to be operational by 2020 at the earliest. Many more states have announced the use of carbon markets in their commitments under the 2015 Paris Agreement on Climate Change. Despite the spread of carbon markets as the primary policy instrument to fight climate change, the empirical evidence to justify this global diffusion is mixed at best. Even worse, pointing at the European experience, academics, policymakers, and market participants are skeptical of carbon market effectiveness. Recent spikes in carbon prices in the EU ETS did little to assuage these concerns.

However, much of this debate revolves around lower than expected prices, especially relative to the social cost of carbon. In this paper, we argue that market prices as such tell us little about how well a carbon market functions. Carbon prices are low, probably because of both oversupply of permits and decreased demand due to decarbonization among regulated polluters. As long as at least some regulated firms see carbon markets as a credible regulatory policy for the future, this is enough for them to move away from carbon-intensive production. This makes carbon reductions compatible with low market prices in the EU ETS. The mechanism we propose here hinges on political commitment to carbon regulation. Interesting testable implications for future research follow from this, both for analyzing how differences in political commitment affect buying and banking decisions within markets and the effectiveness across markets. With a push toward many more carbon markets globally, comparative studies are critical.

Rather than relying on carbon prices, we examine whether the EU ETS has been effective for reducing carbon emissions across Europe. Based on statistical models, we find strong evidence that the EU ETS reduced ${CO}_{2}$ emissions beyond what can be explained by lower emissions during the 2007/2008 financial crisis alone. According to our estimates, EU carbon markets saved cumulative emissions of about 1.2 billion tons ${CO}_{2}$ from 2008 to 2016, or roughly 3.8% relative to total emissions over these years. This does not mean that the EU ETS helped cut worldwide emissions by the same amount because of the possibility of carbon leakage. While we cannot rule out that some emissions were outsourced to countries with weaker regulations, our sector-level results point to substantive reductions in highly immobile industries, such as electricity generation.

Speaking to current debates about the inclusion of additional sectors, such as transport or housing, and the need for active price management, the main takeaway message from this research is that carbon markets can work even when prices are low. For this to be true, however, strong political commitment to continued carbon regulation in the future and increased scarcity in markets is needed. Absent such political will, low prices will do little to decarbonize regulated economies.

## Materials and Methods

### Data.

Our analysis uses the EU Sectoral Emissions Data (EUSED), a newly created dataset (51). It combines information from two sources: emissions data from the EU ETS EUTL (49) and the National Emissions Reported to the United Nations Framework Convention on Climate Change (UNFCCC) and to the EU Greenhouse Gas Monitoring Mechanism (50). A challenge for using the generalized synthetic control is that it requires EU ETS emissions data for years before the EU ETS was launched in 2005. Such data do not exist.

We construct these data in a two-step procedure. First, for years 2005 to 2016, where emissions data are available from both sources, we identify sectors and groups of sectors across the EU ETS and UN data, whose emissions are as close to each other as possible. Second, for the years before 2005, we then use UN emissions for these very same sectors as the values for what EU ETS emissions in the same sector would have looked like. This gives us pretreatment/pre-EU ETS emissions data for sectors which will come under EU ETS regulation after its launch.

As a further complication, the EU ETS and UN data use different definitions of what constitutes a “sector.” The UN follows their Common Reporting Framework (CRF), and the EU data define sectors in terms of EU ETS activities. In matching sectors, we follow official guidelines about the correspondence of CRF categories and EU ETS activities (ref. 50, p. 35ff) and, where discretion applies, match sectors such that their emissions are as close to each other as possible. We construct EUSED sectors by matching as follows: energy, UN category 1.A.1 to EU ETS activities 20/21; metals, UN categories 1.A.2.a, 1.A.2.b, and 2.C to EU ETS activities 22 to 28; minerals, UN categories 1.A.2.f and 2.A to EU ETS activities 29 to 34; chemicals, UN categories 1.A.2.c and 2.B to EU ETS activities 37 to 44; and paper, UN category 1.A.2.d to EU ETS activities 35 and 36. We create two datasets: one that records sector-level emissions and another one that sums up sector-level emissions and records total emissions. We use the latter one for our main analysis.

We also assess the accuracy of our EUSED data. For the 2005 to 2016 years, where EU ETS and UN data are available, we calculate a ratio ρ as the EU ETS emissions over UN emissions, so that ρ=1 would indicate that EU ETS and UN emissions are exactly the same. We show accuracy information by country and by sector (*SI Appendix*, Tables S1 and S2). For the data we use in our main analysis, we achieve very high accuracy of $\bar{\rho }=0.954$ when we average across all countries and $\bar{\rho }=0.972$ when we average across countries and weight by countries’ emissions levels. The data also obtain excellent matches not only in levels but also in trends as an average $R^{2}=0.997$ in country-by-country regressions (through the mean) of EU ETS emissions on UN emissions shows. Further information about the data, a codebook, and R code to construct the data are available from P.B.’s Harvard Dataverse at https://dataverse.harvard.edu/dataverse/eused.

### Generalized Synthetic Control.

We use the generalized synthetic control method (15) to derive estimates for the ATT.

The idea behind synthetic control is straightforward: one can use control units and weight them until they look like the treated units before the treatment is administered (46, 47). Each observation in the control group is weighted according to its ability to bring the (weighted) control group closer to the treatment group.

Ref. 15 combines this basic approach with an interactive fixed effects model (48). This allows us to estimate causal effects with multiple treated units and can also account for unobserved time-varying confounders. In our case, this is important as the 2007/2008 financial crisis had different effects on European economies. These unobserved heterogeneous shocks, which cannot be picked up by simple unit and time fixed effects, are modeled through interactive fixed effects. The generalized synthetic control method conducts dimension reduction prior to reweighting, so, unlike the standard synthetic control approach, weights are not directly interpretable.

To recover ATT estimates, we follow the three-step procedure in ref. 15, with i indexing countries, j indexing sectors, and t indexing time; $t^{ETS}$ denotes treatment year (i.e., 2005 or 2008).

$$\begin{array}{lllll} & \left(1\right) & Y_{ijt} & =X_{it}\gamma +F_{t}\lambda_{i}+\varepsilon_{ijt}, & \text{control group data},\;t=1,\ldots ,T\\ & \left(2\right) & Y_{ijt} & =X_{it}\hat{\gamma }+{\hat{F}}_{t}\lambda_{i}+\eta_{ijt}, & \text{treatment group data,}\;t<t^{ETS}\\ & \left(3\right) & {\hat{Y}}_{ijt} & =X_{it}\hat{\gamma }+{\hat{F}}_{t}{\hat{\lambda }}_{i}, & \text{treatment group data,}\;t\geq t^{ETS} \end{array}$$

where $F_{t}$ are the latent factors (time-varying coefficients) that may or may not interact and $\lambda_{i}$ are their unknown factor loadings (unit-specific intercepts). Our main models are estimated with three latent factors as a result of the model cross-validation procedure that is run as part of the generalized synthetic control algorithm.

The first step recovers $\hat{\gamma }$ and $\hat{F}$. The second step estimates factor loadings, $\hat{\lambda }$, and the third step estimates the counterfactual for treated units in posttreatment periods. The ATT estimator in posttreatment periods is $\hat{ATT}=\frac{1}{N}\left[Y_{ijt}-{\hat{Y}}_{ijt}\right]$ for $t\geq t^{ETS}$.

The linear interactive fixed effects models we estimate are of the following general form:

$${\text{CO}}_{2}{\left(\text{log}\right)}_{ijt}=\tau_{it}{\text{ETS}}_{it}+X_{it}^{\prime }\gamma +F_{t}\lambda_{i}+\varepsilon_{ijt},$$

where $ETS_{it}=\left\{0,1\right\}$ is our binary treatment indicator and $X_{it}$ is a vector of control variables. For all our models, sector j denotes whether total/sector emissions are covered by the EU ETS or not, so j={covered,not covered}. The main analysis uses total emissions, while the sector-level analysis uses sector emissions for four sectors in EUSED: energy, metals, minerals, and chemicals for emissions covered under the EU ETS.

Our main specification includes log(GDP) and log(GDP)^2^ as main variables to the interactive fixed effects model. Results are robust to including the following variables individually or all at once (*SI Appendix*, Table S3): log(GDP per capita), log(Population), log(Renewable electricity production in kwh), and binary carbon tax indicator. The main quantity of interest is $\tau_{it}$.

We use nonparametric bootstrapping with 1,000 runs to generate 95% confidence intervals around the ATT estimates as implemented in the synthetic control method (ref. 15, p. 65).

### Robustness Tests.

In *SI Appendix*, we report several tests that confirm the robustness of our main results. In *SI Appendix*, Fig. S2, we replicate our main results for three different samples: 1) for EU10 countries, 2) for EU15 countries, and 3) for removing one country at a time (leave-one-out-robustness). In *SI Appendix*, Fig. S3, we move the start date of the EU ETS within a ±5-y window around 2008. We find that the decline in emissions which we attribute to the EU ETS does not occur before or after 2008. In *SI Appendix*, Fig. S4, we replicate our analysis for the transport sector, which is not covered under the EU ETS. We do not find a decline in ${CO}_{2}$ emissions, as expected.

### Other Materials.

In *SI Appendix*, Fig. S5, we show absolute and relative ${CO}_{2}$ emission reductions by country, 2008 to 2016. In *SI Appendix*, Fig. S6, we show the treatment status over time. In *SI Appendix*, Fig. S7, we show raw emissions data by country and year.

## Supplementary Material

Supplementary File
