# Supplementary material for: The European Union Emissions Trading System reduced CO2 emissions despite low prices
Source: Proc Natl Acad Sci U S A. 2020 Apr 6;117(16):8804–12. doi: 10.1073/pnas.1918128117 (PMC7183178; doi:10.1073/pnas.1918128117)
Supplement: Supplementary File [file pnas.1918128117.sapp.pdf]

The European Union Emissions Trading System reduced CO_2 emissions despite low prices

— Supplementary Information —

Patrick Bayer* Michaël Aklin†

February 28, 2020

*Corresponding author. School of Government & Public Policy. University of Strathclyde. McCance Building, 16 Richmond Street, Glasgow, G1 1QX, Scotland, UK. Email: patrick.bayer@strath.ac.uk.

†Department of Political Science. University of Pittsburgh. 4815 WWPH, 230 S. Bouquet Street, Pittsburgh, PA 15260, USA. Email: aklin@pitt.edu.

www.pnas.org/cgi/doi/10.1073/pnas.1918128117

S1 Overview

List of Figures

Figure S1 Emission paths by country 8

Figure S2 Different samples 9

Figure S3 Placebo tests (varying timing of treatment) 12

Figure S4 Placebo test (transportation sector) 13

Figure S5 Absolute and relative emission reductions by country, 2008-2016 15

Figure S6 Treatment status 16

Figure S7 Raw emissions data by country, 2005-2016 19

List of Tables

Table S1 Accuracy of EUSED data by country, 2005-2016. 3

Table S2 Accuracy of EUSED data by sector, 2005-2016. 4

Table S3 Results from different model specifications 5

Table S4 Estimated emission reductions by country, 2008-2016 14

S2 Assessment of data quality

ID	Country	ρ average	R^2	ID	Country	ρ average	R^2
1	Austria	0.907	0.999	14	Latvia	0.942	1.000
2	Belgium	0.938	0.997	15	Lithuania	0.873	0.999
3	Cyprus	0.994	1.000	16	Luxembourg	0.848	0.975
4	Czech Republic	0.928	1.000	17	Malta	1.000	0.997
5	Denmark	0.984	0.999	18	Netherlands	0.921	0.999
6	Estonia	0.914	1.000	19	Poland	0.958	1.000
7	Finland	0.959	0.999	20	Portugal	0.970	0.995
8	France	0.950	0.998	21	Slovakia	0.971	1.000
9	Germany	1.03	1.000	22	Slovenia	0.975	0.999
10	Greece	0.949	0.998	23	Spain	0.967	0.992
11	Hungary	0.946	0.997	24	Sweden	0.959	1.000
12	Ireland	1.02	1.000	25	United Kingdom	0.993	0.999
13	Italy	0.962	0.999				

Table S1: Accuracy of EUSED data by country, 2005-2016. For each country, ρ, defined as EU ETS emissions divided by UN emissions, shows how well UN emissions approximate EU ETS emissions (in levels); values of $\rho = 1$ indicate a perfect match. Reported R^2 statistics are from by-country regressions through the mean when regressing EU ETS emissions (dependent variable) on UN emissions (independent variable) to quantify goodness-of-match in terms of trends, not levels. Unweighted average $\bar{\rho} = 0.954$; weighted (by total country emissions) average $\bar{\rho} = 0.972$; average $R^2 = 0.997$.

ID	Sector	ρ average		Mean R^2
		unweighted	weighted	
1	Energy	1.044	1.061	0.998
2	Metals	0.820	0.828	0.940
3	Minerals	0.909	0.903	0.992
4	Chemicals	0.305	0.368	0.710
5	Paper	30.263	1.145	0.907
	Total emissions	0.954	0.972	0.997

Table S2: Accuracy of EUSED data by sector, 2005-2016. For each sector, ρ, defined as EU ETS emissions in this sector divided by UN sectoral emissions, shows how well sectoral UN emissions approximate EU ETS emissions (in levels); values of $\rho = 1$ indicate a perfect match. Unweighted values average by country within sector, and weighted values use each country's sectoral emissions as weights. The reported mean R^2 statistic for each sector comes from by-country regressions through the mean when regressing sectoral EU ETS emissions (dependent variable) on sectoral UN emissions (independent variable) to quantify goodness-of-match in terms of trends, not levels. Match quality for the chemical and paper sectors perform most poorly, while low accuracy in the paper industry is due to data reporting problems.

S3 Alternative model specifications

Model specification	Mean	95% Confidence intervals
Main model		
log(GDP), log(GDP)^2	-11.5%	[-16.9%, -5.4%]
Alternative specifications		
(1) + log(GDP per capita)	-12.1%	[-18.7%, -5.1%]
(2) + log(Population)	-12.0%	[-18.3%, -4.6%]
(3) + log(Renewable electricity production in kwh)	-7.7%	[-14.4%, -2.5%]
(4) + carbon tax indicator	-11.5%	[-16.7%, -5.3%]
(5) full model	-9.3%	[-16.1%, -2.8%]

Table S3: Summary of average treatment effect on the treated (ATT) and 95% confidence intervals for different model specifications, 2008-2016. All models include log(GDP) and log(GDP)^2 as well as interactive fixed effects in the main model plus the additional variable shown in the first column. The full model includes all variables listed.

S4 Results by country

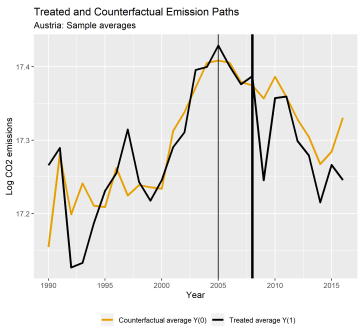

(a) Austria

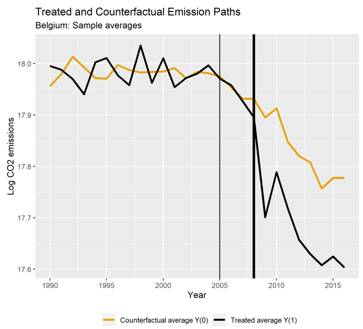

(b) Belgium

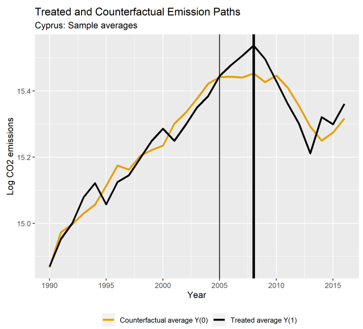

(c) Cyprus

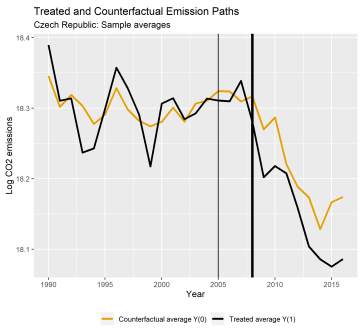

(d) Czech Republic

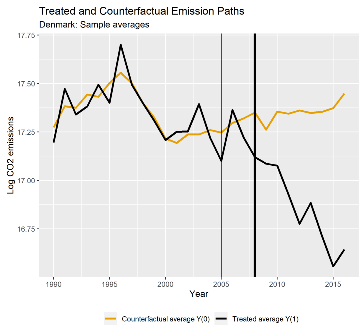

(e) Denmark

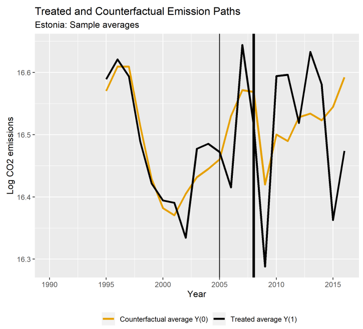

(f) Estonia

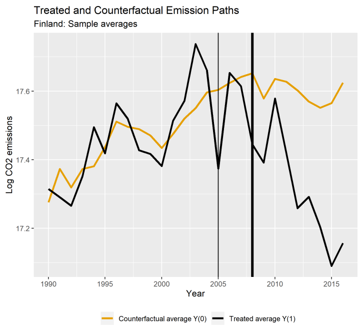

(g) Finland

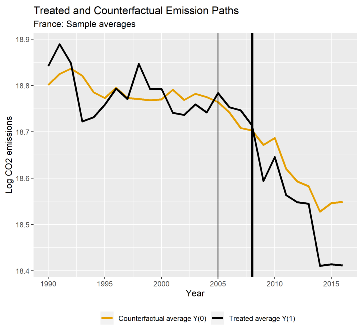

(h) France

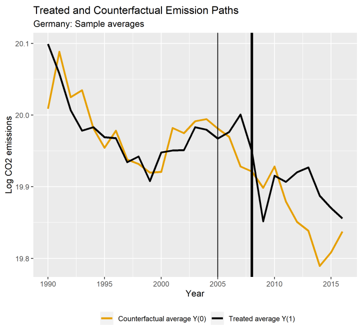

(i) Germany

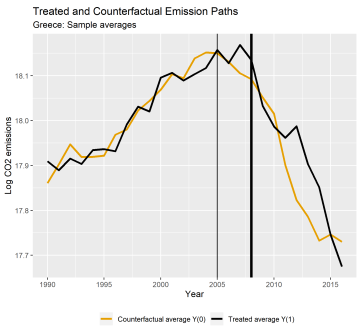

(j) Greece

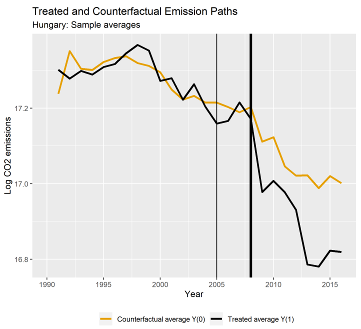

(k) Hungary

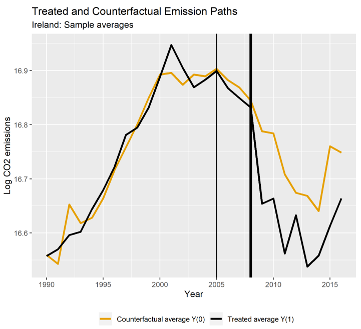

(l) Ireland

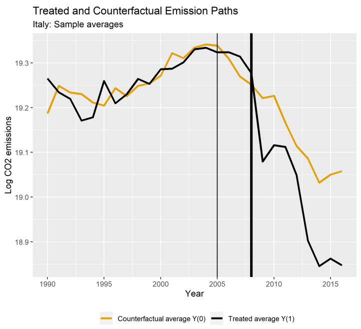

(m) Italy

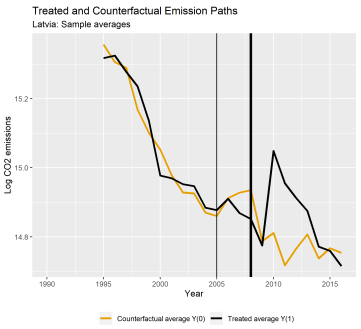

(n) Latvia

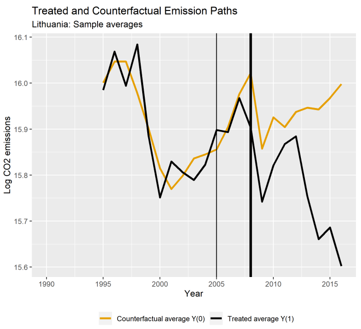

(o) Lithuania

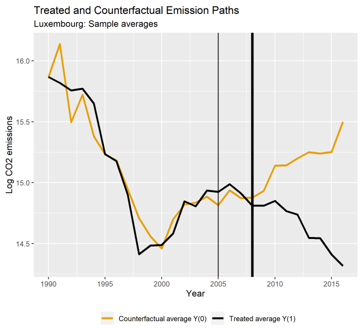

(p) Luxembourg

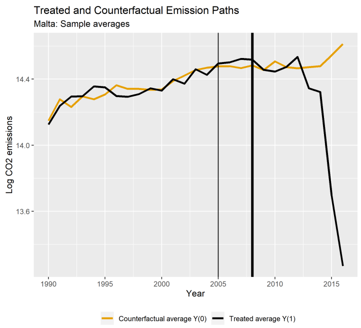

(q) Malta

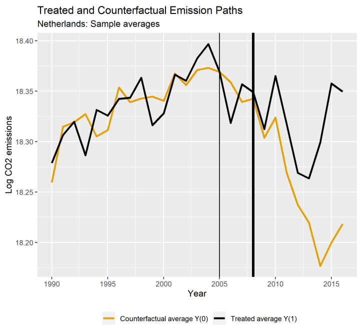

(r) Netherlands

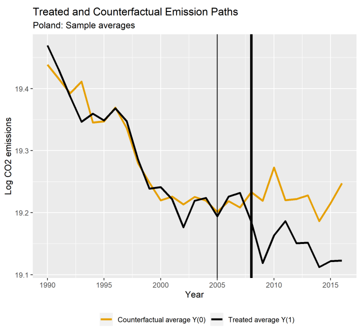

(s) Poland

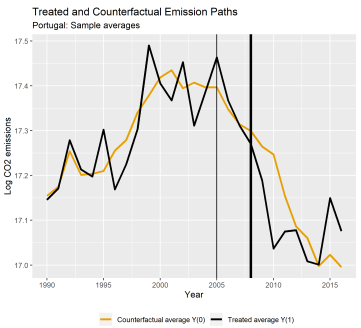

(t) Portugal

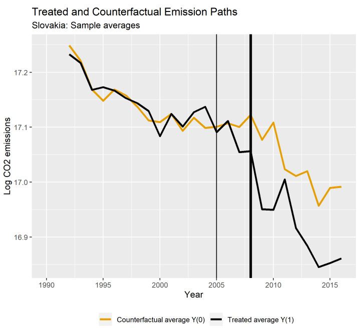

(u) Slovakia

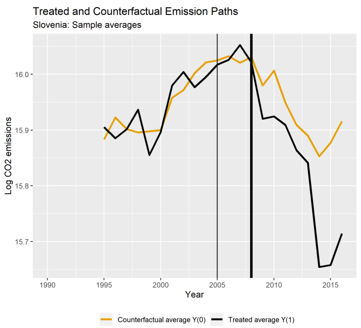

(v) Slovenia

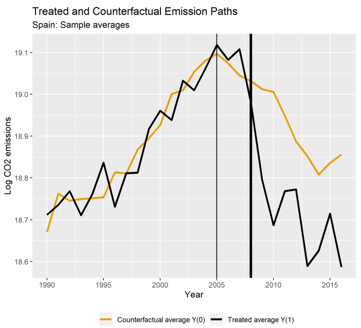

(w) Spain

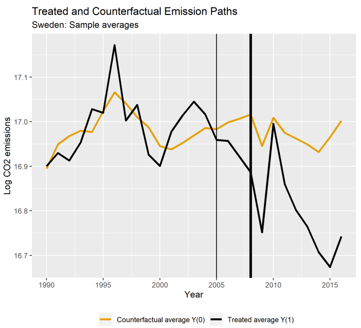

(x) Sweden

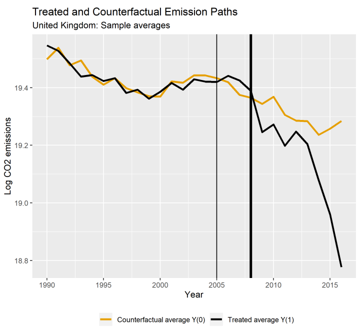

(y) United Kingdom

Figure S1: Emission paths of counterfactual and treated emissions by country.

S5 Results with different samples

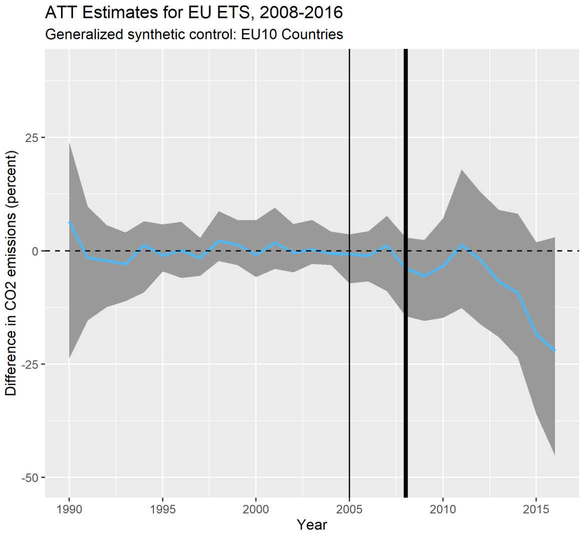

(a) EU10 sample

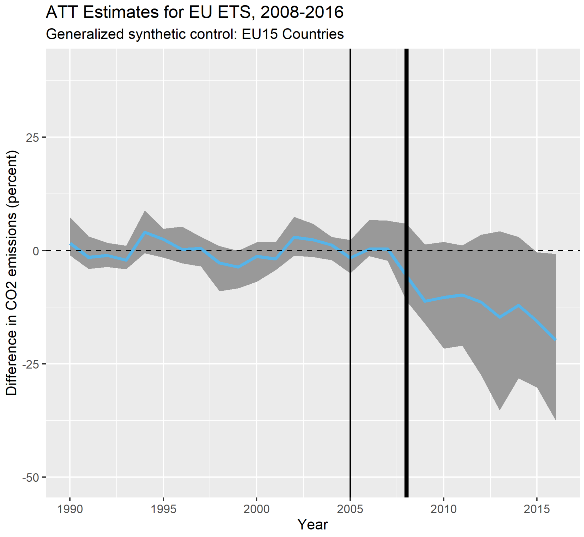

(b) EU15 sample

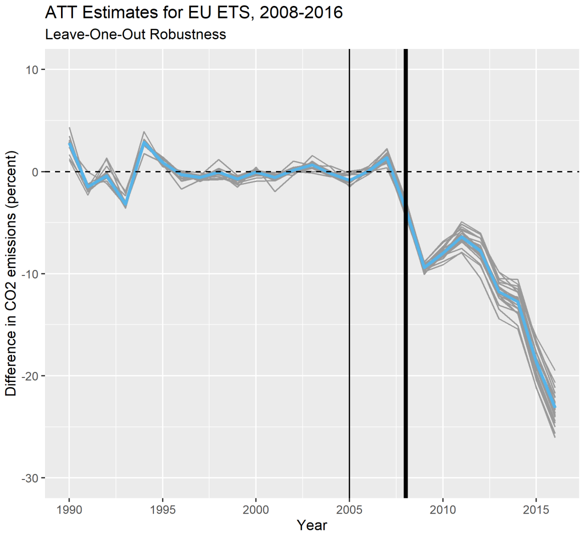

(c) Leave-one-out robustness

Figure S2: Results using other samples: EU10 sample only includes EU10 countries; EU15 sample only includes EU15 countries; 'Leave-one-out' sample replicates the main results, sequentially leaving out one country at a time. The blue line represents our original estimate for the average treatment effect on the treated (ATT), while the gray line shows the new ATT estimates when dropping one country at a time. This robustness check shows that the main results are not driven by events or policies in a single country.

S6 Placebo test: Varying treatment year

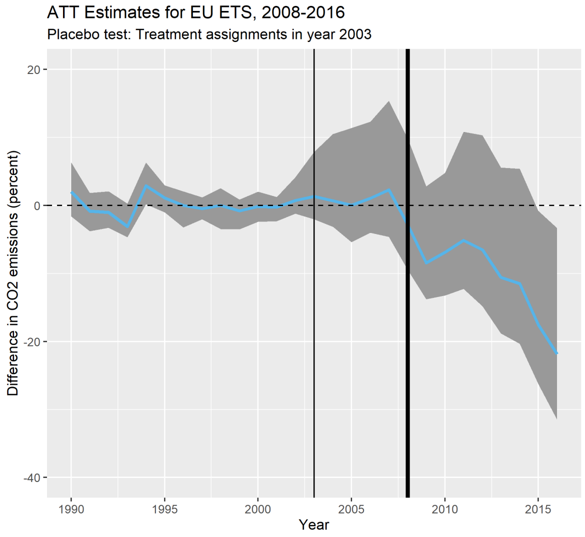

(a) 2003

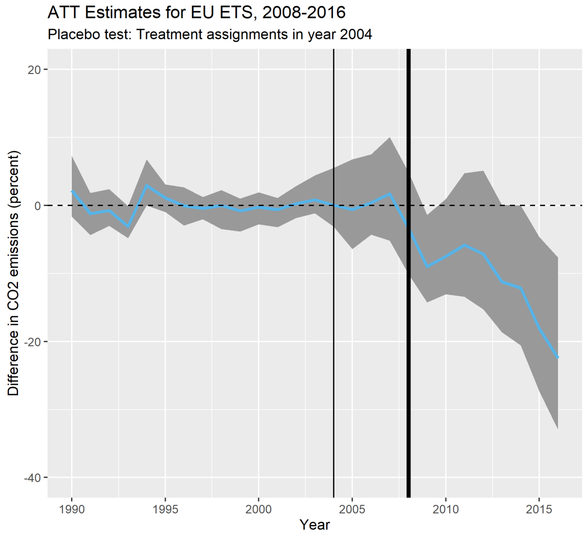

(b) 2004

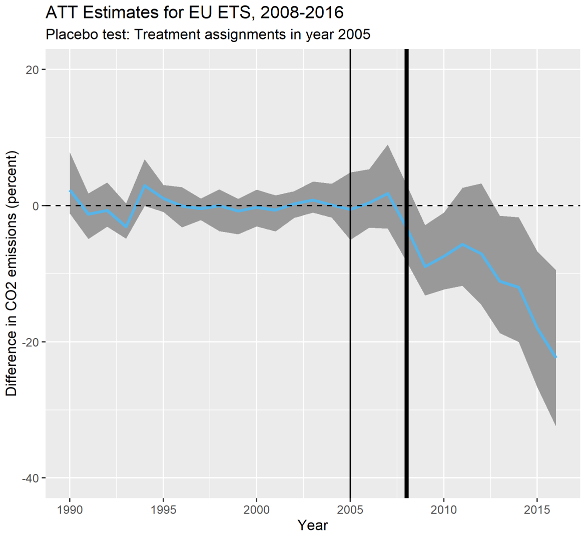

(c) 2005

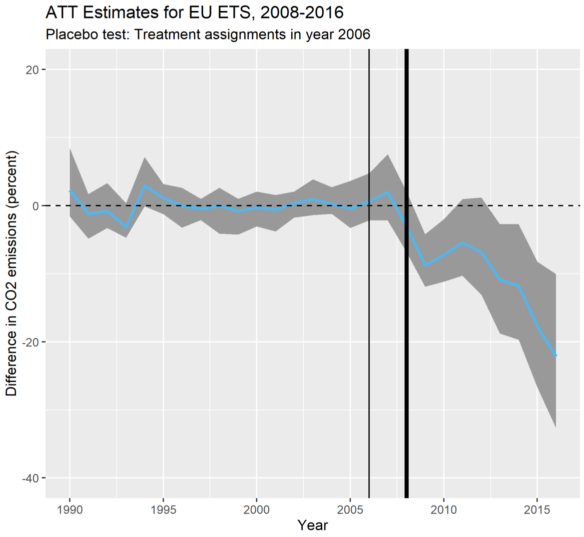

(d) 2006

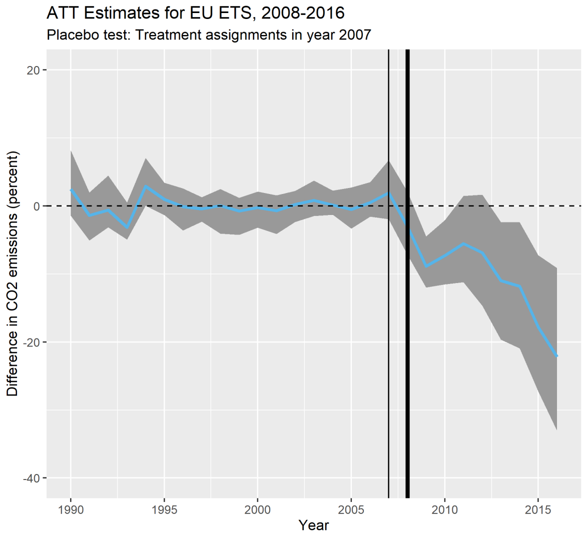

(e) 2007

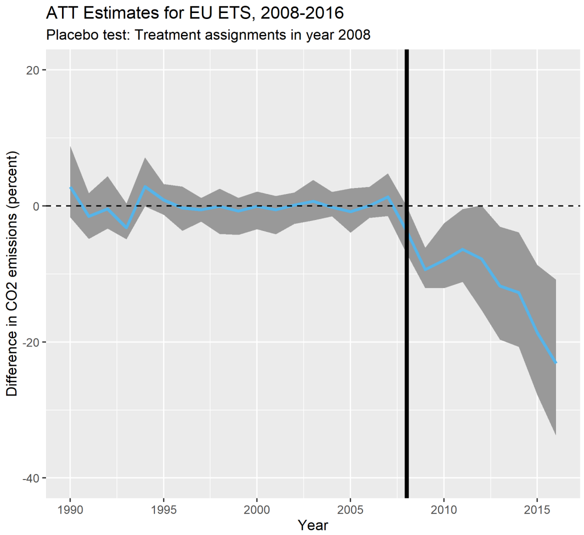

(f) 2008

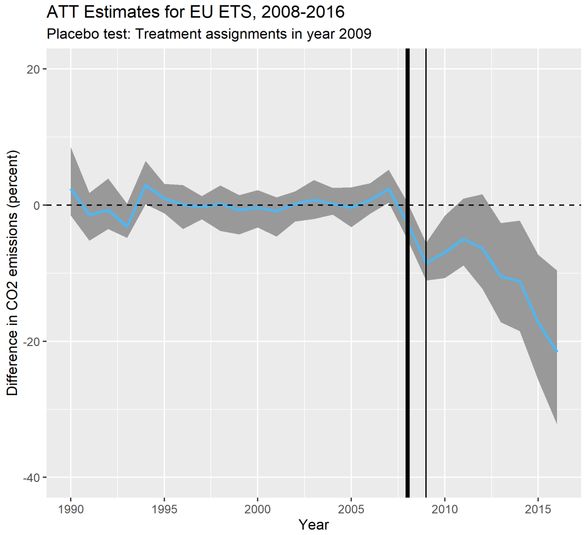

(g) 2009

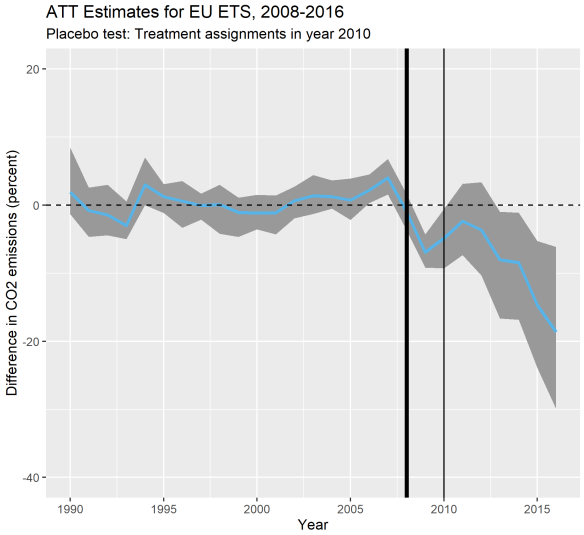

(h) 2010

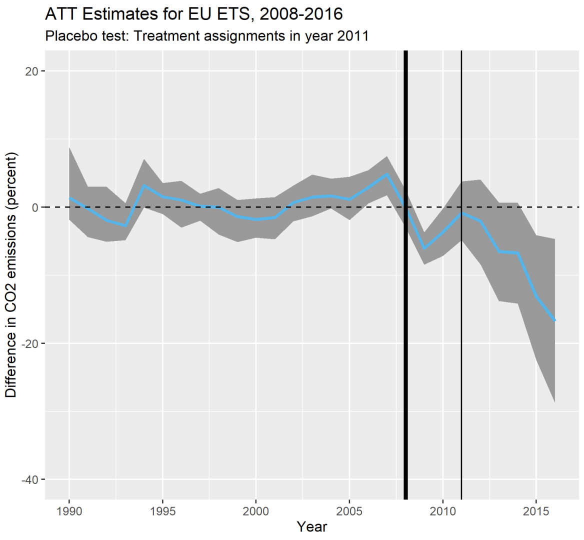

(i) 2011

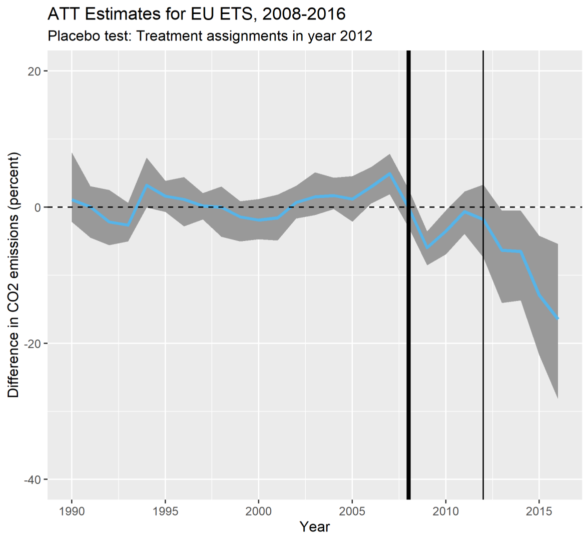

(j) 2012

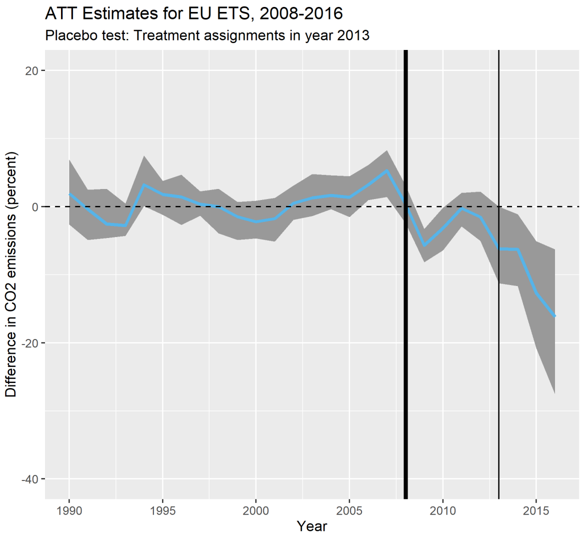

(k) 2013

Figure S3: Placebo tests. We simulate the effect of the EU ETS by changing the start of the treatment year (thin straight line) in a $\pm$5-year window around 2008 when the EU ETS was launched. The robustness check shows that the ATT breaks off from the zero line of no effect almost exactly in 2008. This indicates that the main results are not driven by other policies adopted before or after the start of the second trading period of the EU ETS in 2008.

S7 Placebo test: Effect for non-ETS transport sector

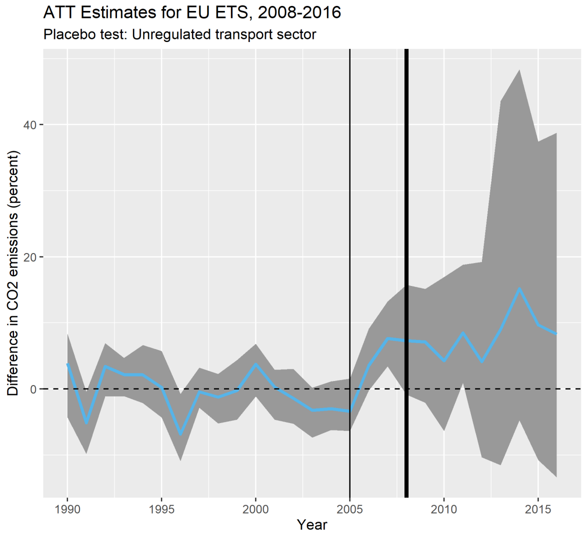

Figure S4: Placebo test. We simulate the effect of the EU ETS on the transportation sector, which is *not* covered under the EU ETS. We therefore expect no decline in CO_2 emissions after 2008, which is exactly what we find.

S8 Estimated emission reductions from EU ETS

	Country	Difference Y(1)-Y(0)	ETS emissions	Total emissions	ETS emissions (%)	Total emissions (%)
1	Austria	-10.9	292.5	617.8	-3.7	-1.8
2	Belgium	-66.5	436.1	946.3	-15.2	-7.0
3	Cyprus	0.5	42.7	67.5	1.2	0.8
4	Czech Republic	-39.7	693.6	1003.4	-5.7	-4.0
5	Denmark	-116.9	193.5	385.4	-60.4	-30.3
6	Estonia	-1.4	133.6	160.5	-1.1	-0.9
7	Finland	-95.4	301.1	478.0	-31.7	-20.0
8	France	-70.6	1017.8	3270.3	-6.9	-2.2
9	Germany	141.0	3946.2	7329.2	3.6	1.9
10	Greece	23.8	550.4	805.5	4.3	3.0
11	Hungary	-29.2	202.1	440.4	-14.4	-6.6
12	Ireland	-15.7	151.5	359.5	-10.3	-4.4
13	Italy	-202.9	1640.0	3532.0	-12.4	-5.7
14	Latvia	1.7	25.5	69.1	6.6	2.4
15	Lithuania	-11.9	63.8	122.1	-18.7	-9.8
16	Luxembourg	-14.4	20.9	93.0	-69.0	-15.5
17	Malta	-3.2	14.6	21.6	-22.2	-15.0
18	Netherlands	51.2	814.7	1514.6	6.3	3.4
19	Poland	-157.1	1859.5	2886.0	-8.4	-5.4
20	Portugal	-7.3	240.7	470.1	-3.1	-1.6
21	Slovakia	-23.0	202.1	328.9	-11.4	-7.0
22	Slovenia	-6.8	68.3	139.7	-9.9	-4.9
23	Spain	-252.4	1228.3	2522.1	-20.5	-10.0
24	Sweden	-33.2	178.5	420.1	-18.6	-7.9
25	United Kingdom	-279.2	1898.5	4217.9	-14.7	-6.6
	Total (EU25)	-1219.5	16216.5	32202.0	-7.5	-3.8

Table S4: Emission reductions in million tons CO_2 by country, 2008-2016.

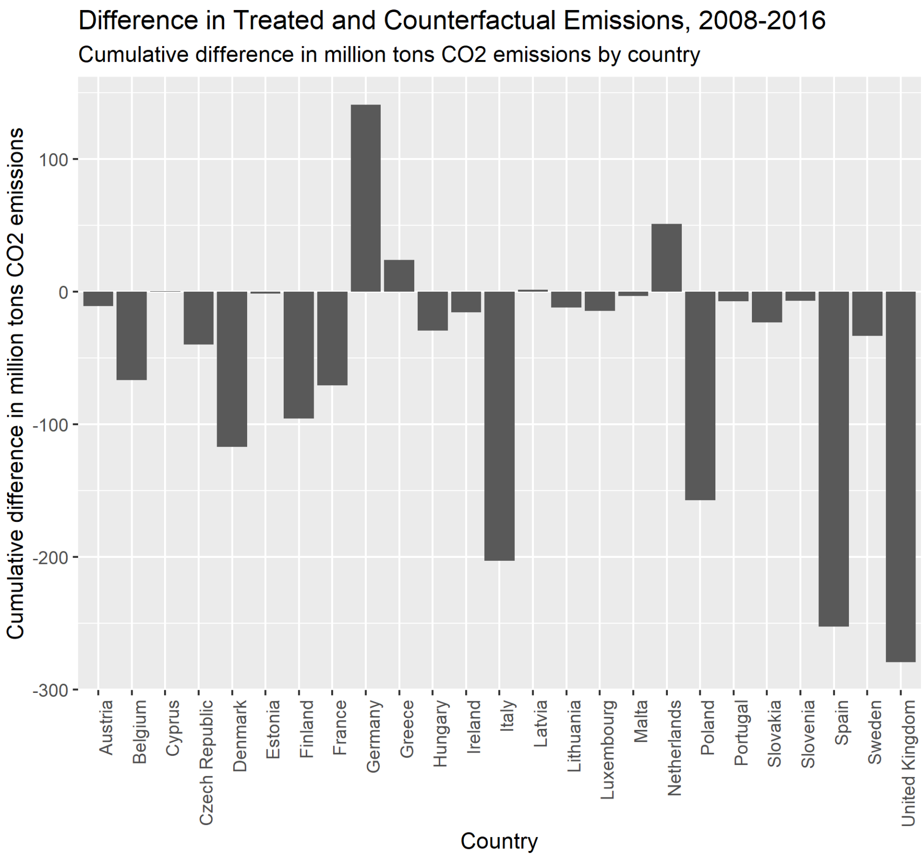

(a) Absolute change in million tons CO_2 emissions

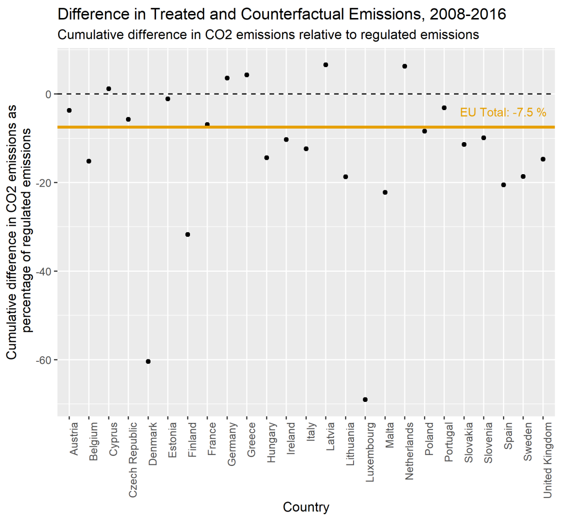

(b) Emission reductions relative to CO_2 emissions covered under the EU ETS

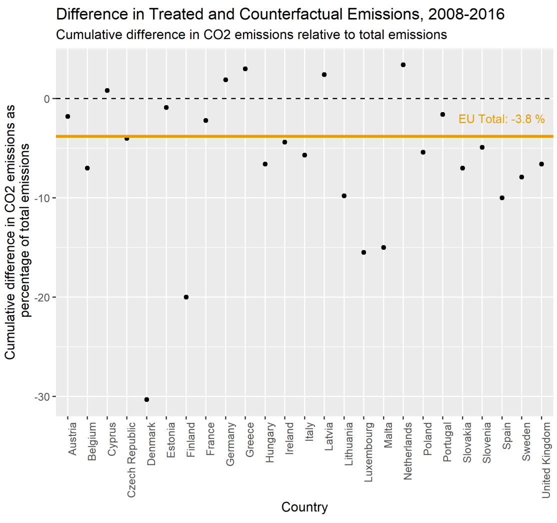

(c) Emission reductions relative to total CO_2 emissions

Figure S5: Treatment effect of the EU ETS by country, 2008-2016. The change is reported in absolute reductions of million tons CO_2 emissions (top) and as reductions relative to both CO_2 emissions covered under the EU ETS (bottom left) and total CO_2 emissions (bottom right). Reductions are summed over years 2008-2016.

S9 Treatment status

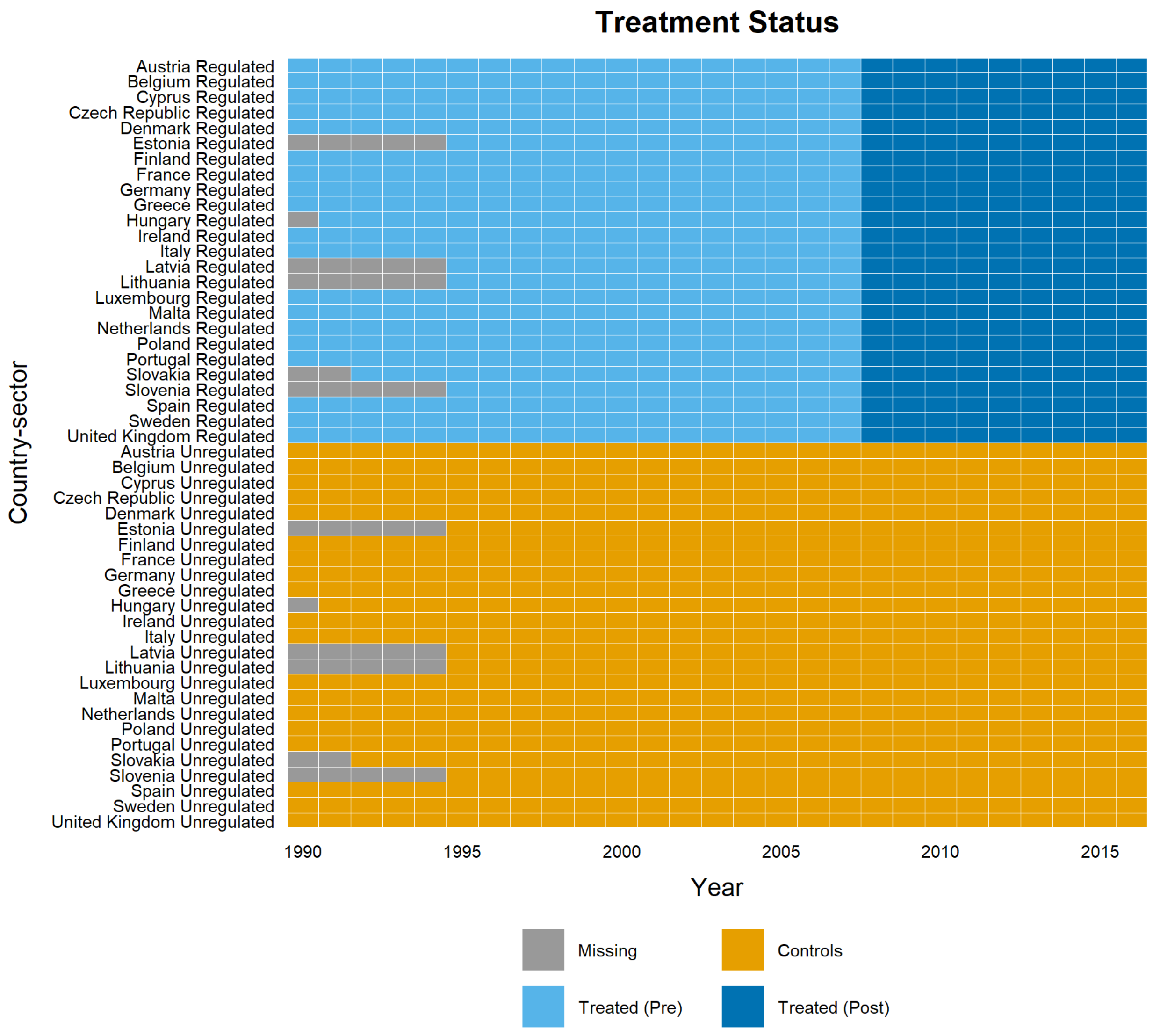

Figure S6: Description of treatment status. Tiles in yellow indicate emissions from non-ETS sectors by country-year, which are used to construct the counterfactual with the help of the generalized synthetic control. Tiles in blue indicate emissions from ETS sectors by country-year, separately for pre-treatment, pre-EU ETS years (light blue) and post-treatment, post-EU ETS years (dark blue).

S10 Raw emissions data by country

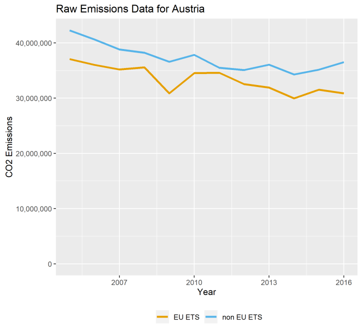

(a) Austria

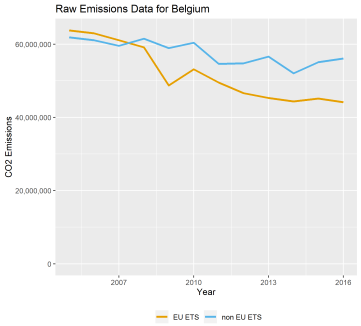

(b) Belgium

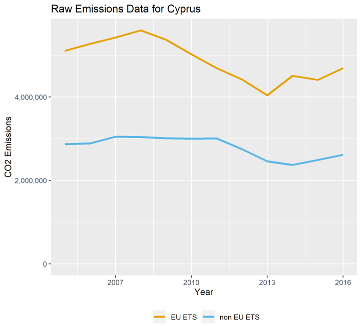

(c) Cyprus

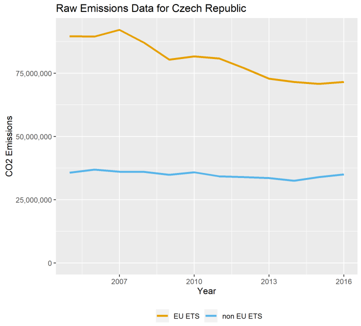

(d) Czech Republic

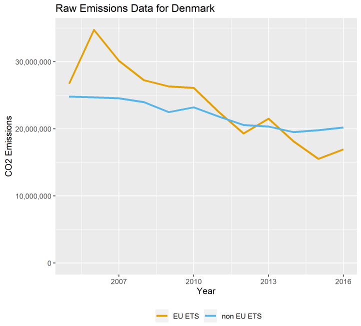

(e) Denmark

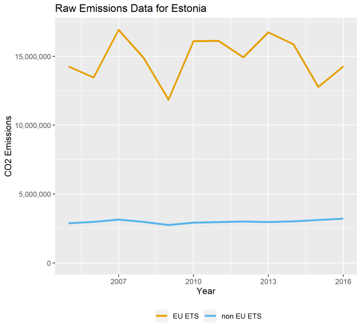

(f) Estonia

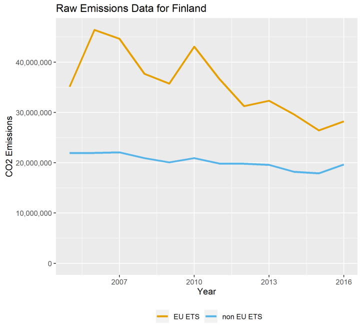

(g) Finland

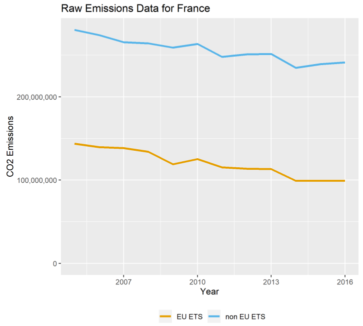

(h) France

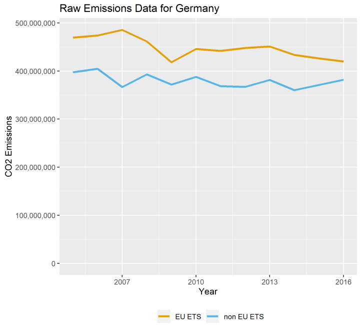

(i) Germany

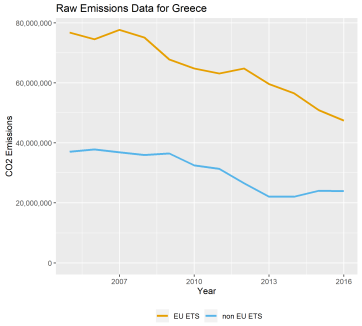

(j) Greece

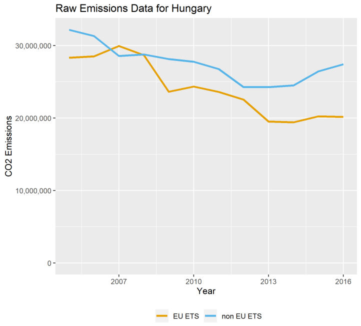

(k) Hungary

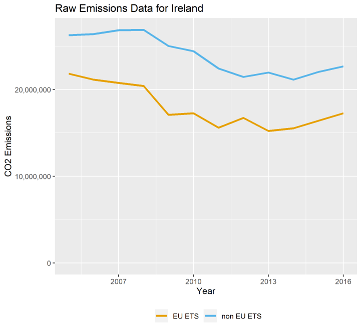

(l) Ireland

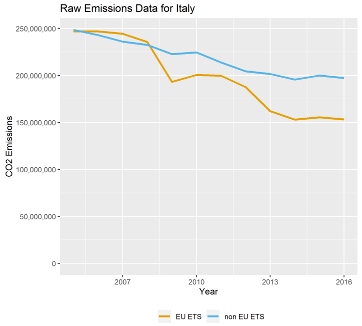

(m) Italy

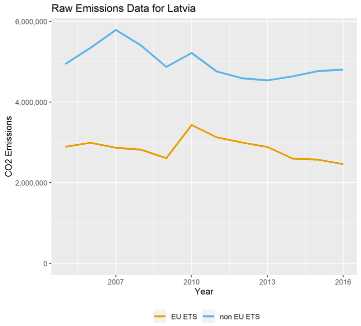

(n) Latvia

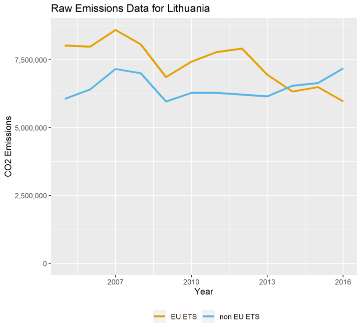

(o) Lithuania

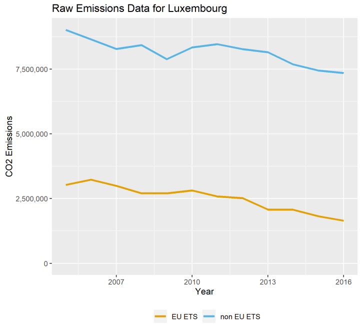

(p) Luxembourg

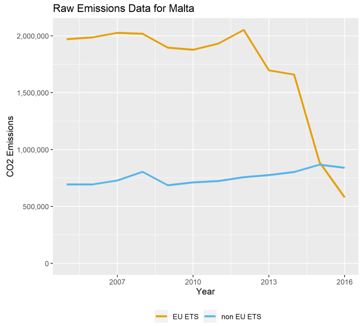

(q) Malta

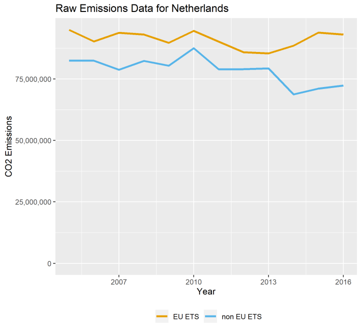

(r) Netherlands

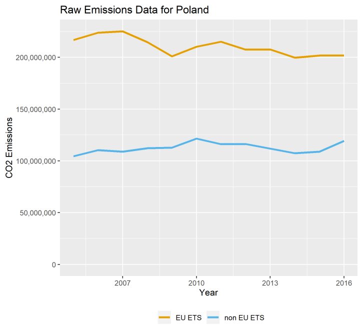

(s) Poland

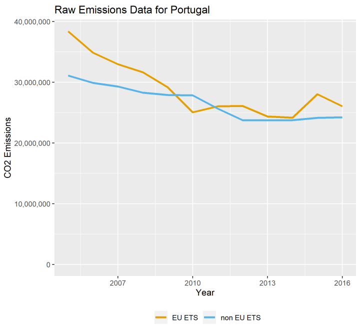

(t) Portugal

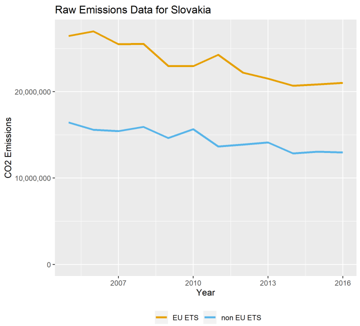

(u) Slovakia

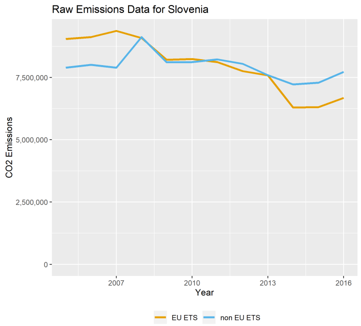

(v) Slovenia

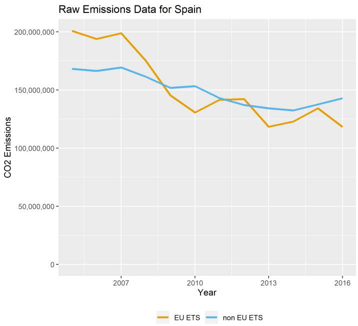

(w) Spain

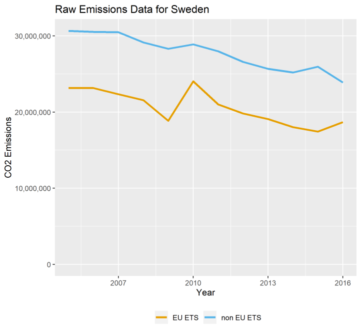

(x) Sweden

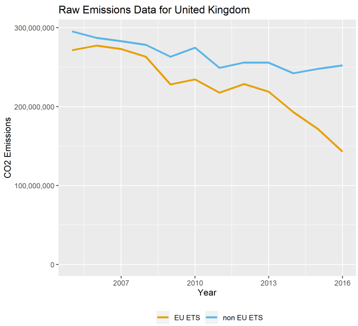

(y) United Kingdom

Figure S7: Raw emissions data by country, 2005-2016.
